# Care of the Hands

**Published:** 1893-01

**Authors:** 


### CARE OF THE HANDS.

It is universally acknowledged that a soft, white hand, with well shaped nails, adds greatly to the charm of every woman, be she young or old, but there are many who, from choice or necessity, busy themselves actively in their houses or about their gardens, and consequently have considerable difficulty in keeping their hands in a presentable state. Of course, if gloves are worn, they preserve the skin from stains and friction, but some persons—the writer is one—find it both difficult and disagreeable to do any kind of work satisfactorily, save with bare fingers.

Now, a little care and attention bestowed on the hands regularly will enable those of the most active housewife to be kept as daintily as the most scrupulous person could desire. Supposing that the skin has been allowed to become coarse and rough from long neglect, the first thing to be done in order to get it into proper condition is to wash the hands every night in thin oatmeal gruel—this must be made fresh every other day—drying them thoroughly, and smearing them afterwards with a paste made of equal parts of white wax, honey, cocoa-butter and almond oil stirred together in a fire-proof china jar on a stove or hot-plate ; or one made of 2 oz. refined honey, 1 dessert-spoonful lemon juice, 8 drops of oil of bitter almonds, the whites of two eggs and enough fine oatmeal to make the mixture of the required consistence. A pair of old white kid or thin wash leather gloves, very loose fitting and with the palms cut out for the sake of ventilation, should be worn for a few nights. When this treatment has had the desired effect, and the hands have become soft and white, they can be kept so by rubbing in a little of either of the pastes for which the recipes are given above two or three times a week, or rather oftener in the winter, always taking care to dry the hands very thoroughly, and to rub a little lemon on them after washing.

Glycerine should not be used under any circumstances, as it clogs the pores and gives the skin a yellow, unhealthy appearance, but if the hands are much chapped a little lanoline (wool fat) will be found beneficial. To get the nails in order, the tips of the fingers should be soaked in hot water, to which a little borax has been added, and the nails carefully trimmed to a nice shape, pushing back the skin round the base with a small bone instrument sold for the purpose, and finally polishing them with a bit of chamois leather. Afterwards the nails should be frequently filed down to obviate the necessity of cutting, and if they seem at all inclined to break they should be well greased at night. Many people are much troubled with excessive perspiration of the palms, and although it is not well to check the free action of the pores in any part of the body, still a small portion of this powder can be rubbed in occasionally when the annoyance is unusually great : 2 oz. of fullers' earth, ½ oz. bismuth, 2 drams tannin, finely powered and mixed.

